# Assessing HDL Metabolism in Subjects with Elevated Levels of HDL Cholesterol and Coronary Artery Disease

**DOI:** 10.3390/molecules26226862

**Published:** 2021-11-14

**Authors:** William Hancock-Cerutti, John S. Millar, Silvia Valentini, Jason Liu, Jeffrey T. Billheimer, Daniel J. Rader, Marina Cuchel

**Affiliations:** Division of Translational Medicine and Human Genetics, Perelman School of Medicine University of Pennsylvania, Philadelphia, PA 19104, USA; william.hancock-cerutti@yale.edu (W.H.-C.); jsmillar@pennmedicine.upenn.edu (J.S.M.); dottvalentini@gmail.com (S.V.); jasonyliumd@gmail.com (J.L.); billheij@pennmedicine.upenn.edu (J.T.B.); rader@pennmedicine.upenn.edu (D.J.R.)

**Keywords:** hyperalphalipoprotenemia, lipoprotein metabolism, cholesterol efflux, coronary artery disease

## Abstract

High-density lipoprotein cholesterol (HDL-C) is thought to be atheroprotective yet some patients with elevated HDL-C levels develop cardiovascular disease, possibly due to the presence of dysfunctional HDL. We aimed to assess the metabolic fate of circulating HDL particles in patients with high HDL-C with and without coronary artery disease (CAD) using in vivo dual labeling of its cholesterol and protein moieties. We measured HDL apolipoprotein (apo) A-I, apoA-II, free cholesterol (FC), and cholesteryl ester (CE) kinetics using stable isotope-labeled tracers (D_3_-leucine and ^13^C_2_-acetate) as well as ex vivo cholesterol efflux to HDL in subjects with (*n* = 6) and without (*n* = 6) CAD that had HDL-C levels >90th percentile. Healthy controls with HDL-C within the normal range (*n* = 6) who underwent the same procedures were used as the reference. Subjects with high HDL-C with and without CAD had similar plasma lipid levels and similar apoA-I, apoA-II, HDL FC, and CE pool sizes with no significant differences in fractional clearance rates (FCRs) or production rates (PRs) of these components between groups. Subjects with high HDL-C with and without CAD also had similar basal and cAMP-stimulated ex vivo cholesterol efflux to HDL. When all subjects were considered (*n* = 18), unstimulated non-ABCA1-mediated efflux (but not ABCA1-specific efflux) was correlated positively with apoA-I production (r = 0.552, *p* = 0.017) and HDL FC and CE pool sizes, and negatively with the fractional clearance rate of FC (r = −0.759, *p* = 4.1 × 10^−4^) and CE (r = −0.652, *p* = 4.57 × 10^−3^). Our data are consistent with the concept that ex vivo non-ABCA1 efflux capacity may correlate with slower in vivo turnover of HDL cholesterol moieties. The use of a dual labeling protocol provided for the first time the opportunity to assess the association of ex vivo cholesterol efflux capacity with in vivo HDL cholesterol metabolic parameters.

## 1. Introduction

Despite numerous epidemiologic studies indicating a strong inverse association of high-density lipoprotein (HDL) cholesterol (C) and apolipoprotein (apo) A-I levels with the incidence of coronary artery disease (CAD) [1,2,3], and the finding that low HDL-C levels are frequently found in subjects with CAD [4], the causality of the relationship between HDL-C and CAD has been brought into question by several recent reports. Human genetic studies [5,6], Mendelian randomization studies [7,8,9], and recent clinical trials [10,11,12] have cast doubt on the benefit of HDL-C in protecting from cardiovascular disease and fueled a paradigm shift towards the focus on HDL function rather than HDL-C mass. HDL particles are emerging as a heterogeneous group of particles with multiple functions beyond their well-recognized role in atheroprotection [13,14,15,16].

In the context of atherosclerosis, the main protective function of HDL is its role as a dynamic molecular complex that promotes reverse cholesterol transport (RCT), i.e., the transport of excess cholesterol from peripheral cells back to the liver, to be excreted into bile [17,18]. Supporting the importance of this function are preclinical studies examining the atheroprotection of HDL in the context of apoA-I overexpression [19,20], direct infusion of HDL particles or their mimetics [21], and the use of molecules that enhance cholesterol efflux onto HDL, such as liver X receptor (LXR) agonists [22], and numerous studies assessing in vivo reverse cholesterol transport in several animal models [23]. In contrast, animals with high HDL-C levels due to a delayed HDL catabolism and delayed RCT, such as SR-BI knockout (KO) mice, are more prone to atherosclerotic lesions as compared with controls [24,25,26]. Interestingly, individuals with high HDL-C due to loss of function mutations in *SCARB1*, the gene that encodes the scavenger receptor class B type 1 (SR-BI) protein, were also at increased risk of CAD [27]. Furthermore, numerous studies have demonstrated that a measure of the first step of RCT, namely cholesterol efflux capacity, is associated with beneficial CAD outcomes [18,28,29]. However, these studies only offer a partial view of the complex HDL metabolic pathways in vivo.

In vivo metabolic tracer studies have been instrumental in advancing our understanding of HDL metabolism in health and disease in humans [30]. Previous studies using radiolabeled tracers have shown that high HDL-C can result from an increased production of apoA-I as well as delayed apoA-I catabolism [31,32]. More recent studies, performed using endogenous labeling of apolipoproteins with stable isotopes, have helped identify HDL subspecies that are actively expanding in sizes and cleared more or less rapidly depending on their apolipoprotein composition [33,34]. Although these kinetics studies are advancing our understanding of the role of the apolipoprotein moiety in determining the fate of HDL particles, they provided limited information on the metabolic fate of their cholesterol cargo. To address this gap, we performed a feasibility study using two different metabolic tracers, one labeling the protein and the other the cholesterol moiety of HDL, in a sample of subjects with and without CAD that had HDL cholesterol levels > 90th percentile.

## 2. Results

### 2.1. Demographic Data, Lipids, and Apolipoproteins

A total of 18 subjects were recruited for the study. These consisted of six normolipidemic subjects that were enrolled to help finalize the protocol, six subjects with high HDL with CAD, and six healthy subjects with high HDL without CAD (Table 1). Per protocol, subjects in the high HDL-C groups were well matched for age, sex, body mass index (BMI), triglyceride, low-density lipoprotein cholesterol (LDL-C), and apoB levels. In addition, by design, subjects in the high HDL-C groups with and without CAD were matched for total cholesterol, HDL-C, and apoA-I. Normolipidemic subjects, used as the reference group, had total cholesterol and LDL-C levels that were similar to those in subjects in the high HDL-C groups with and without CAD while having significantly lower HDL-C and apoA-I levels. No adverse events associated with the study procedures were reported.

### 2.2. Apolipoprotein Kinetics

**Figure 1:**
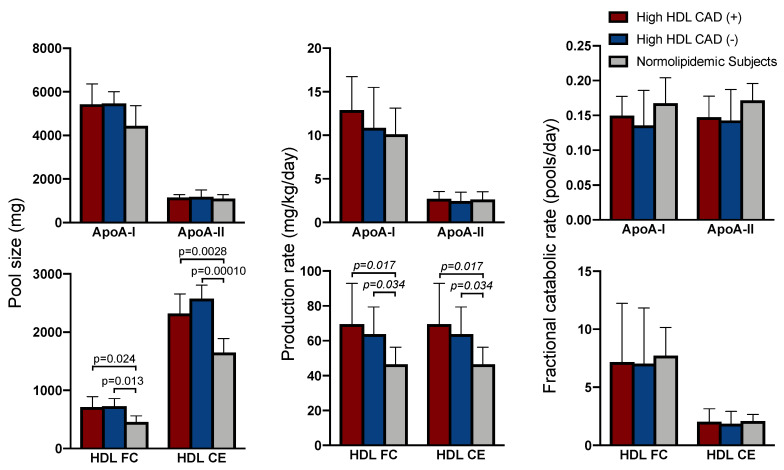
Pool size (PS), production rate (PR), and fractional catabolic rate (FCR) of HDL apoA-I, apoA-II, free cholesterol (FC), and cholesteryl ester (CE). Data are shown as the mean. Error bars represent the standard deviation. *n* = 6 for each group. Differences among groups were determined using ANOVA followed by a Tukey-HSD post-hoc test for variables with a normal distribution and the Kruskal–Wallis test followed by a post-hoc Dunn’s test for variables with a non-normal distribution.

The high HDL subjects with and without CAD tended to have a higher circulating apoA-I pool size (PS) (i.e., total amount circulating in blood) and a similar apoA-II PS as compared to subjects with normal HDL-C levels (Figure 1). The high HDL-C subjects also had apoA-I and apoA-II production rates (PR) and fractional catabolic rates (FCR) that were similar to control subjects with normal HDL-C levels. There were no significant differences in the apoA-I or apoA-II PS or in the apoA-I and apoA-II FCRs and PRs between high HDL subjects with and without CAD.

Since triglyceride-rich lipoproteins influence the risk of cardiovascular disease, we also examined the kinetics of VLDL apoB100 in these subjects. Both groups of subjects with high HDL-C had a VLDL apoB PS that was similar to that from subjects with normal HDL-C levels (Table 2). The subjects with high HDL-C had a lower VLDL apoB PR than normolipidemic subjects while the VLDL apoB FCR in subjects with high HDL-C was similar. There was no significant difference in the VLDL apoB100 PS between the high HDL-C subjects with and without CAD. There were also no significant differences in the VLDL apoB100 PR or FCR between the high HDL-C subjects with and without CAD.

### 2.3. HDL Cholesterol Kinetics

HDL CE and FC PS were significantly higher in subjects with high HDL-C with and without CAD as compared to subjects with normal HDL-C levels (Figure 1). These differences are attributable to higher HDL FC and CE PR than in subjects with normal HDL-C levels (Figure 1). There was no difference among the high HDL-C subjects and normolipidemic subjects in the HDL FC or CE FCR. When comparing high HDL-C subjects with and without CAD, there were no differences in any of the kinetic parameters tested.

### 2.4. Cholesterol Efflux

Both groups of subjects with high HDL-C tended to have higher total efflux and non-ABCA1-mediated efflux than normal control subjects, although this was not statistically significant (*p* = 0.12 and *p* = 0.09, respectively; Table 3). When we combined both groups of high HDL-C subjects (*n* = 12) and compared them to the normolipidemic subjects in a post-hoc analysis, we found that cholesterol efflux in the absence of cAMP (unstimulated, non-ABCA1-mediated efflux) was significantly increased in the high HDL-C subjects (5.25 ± 1.48 vs. 3.88 ± 0.87, *p*-value = 0.041). ABCA1-mediated efflux in subjects with high HDL-C was similar to that measured in subjects with normal HDL-C levels. High HDL-C subjects with or without CAD were similar in their total efflux and ABCA1-mediated and non-ABCA1-mediated efflux capacities.

### 2.5. Correlations between HDL Apolipoprotein and Cholesterol Kinetics and Efflux

Next, we performed an exploratory analysis by conducting correlations on the combined groups of subjects to examine the relationships between HDL apolipoprotein kinetics, HDL cholesterol kinetics, and HDL cholesterol efflux capacity (Table 4, See Supplementary Materials, Figure S1). The apoA-I PS and PR were significantly correlated with the non-ABCA1-mediated efflux capacity. There were significant correlations between the HDL FC and CE PS and the total and non-ABCA1-mediated efflux capacity. On the other hand, the HDL FC and CE FCRs were significantly inversely correlated with both the total cholesterol efflux and non-ABCA1-medated efflux capacities. There were no significant correlations between any of the apolipoprotein and cholesterol kinetics parameters and ABCA1-mediated efflux capacity.

## 3. Discussion

This study demonstrates the feasibility of using in vivo dual labeling with metabolic tracers to simultaneously study the metabolic fate of the protein and cholesterol moieties of HDL. This approach is of relevance to understand the catabolism of the HDL cholesterol cargo that, contrary to that of LDL, differs from the catabolism of its main protein, apoA-I.

The phenotypic profile of high HDL-C, normal plasma triglyceride and LDL-C levels, and normal body weight and blood pressure has been seen as beneficial when assessing cardiovascular risk. Low levels of HDL-C are associated with increased risk of cardiovascular disease while higher levels of HDL-C have been historically associated with longevity [2,4,35]. Yet, patients with high HDL-C levels may still develop cardiovascular disease, suggesting the presence of dysfunctional HDL. One of the main functions of HDL is to promote reverse cholesterol transport and impairment of this process may result in a slower than normal transport of cholesterol through the reverse cholesterol transport pathway [36,37]. A methodologic approach that allows the investigation of the HDL cholesterol flux in vivo can help us better understand the causal role of HDL function in determining cardiovascular disease risk.

The current study compared HDL function in patients with high HDL-C with and without cardiovascular disease. In these well-matched groups of subjects, there were no significant differences in plasma lipid levels, ex vivo cholesterol efflux, or HDL apoA-I production or clearance. In addition, there were no significant differences in the flux of cholesterol into HDL (measured in vivo as HDL FC PR) or in its clearance out of HDL (measured in vivo as HDL FC and CE FCR) HDL. However, the number of subjects was limited. Thus, although these results may suggest that the disparity of CAD burden between the two groups with high HDL is not explained by differences in HDL apolipoprotein and cholesterol kinetics, it is possible that the limited sample size, due to the rarity of patients with the combined high HDL-C and CAD phenotype and the relatively high time-commitment and invasiveness of the in vivo kinetic study, may explain the absence of significant differences.

We previously reported that subjects with high HDL-C and CAD have lower ex vivo cholesterol efflux than high HDL-C subjects without CAD [38]. This was attributed to reduced HDL phospholipid in the CAD-positive group. We have also reported differences in the phosphosphingolipidome between high HDL-C subjects with and without CAD that could contribute for the observed differences in cholesterol efflux capacity [39]. The magnitude of this difference in cholesterol efflux capacity was 7%, which would be unlikely to be detected in the current study due to the limited sample size. Despite this limitation, it is important to note that in previous studies, the ex vivo cholesterol efflux measurement in subjects with high HDL-C and CAD is considerably greater than that measured for subjects with normal HDL levels [38,39], similar to our data. This indicates that despite a modest reduction in the HDL cholesterol efflux capacity as compared to subjects with high HDL-C and no CAD, these subjects still have a high cholesterol efflux capacity compared to subjects with normal HDL levels, when measured using the commonly used ex vivo assay [38]. In accordance with these results, we found increased unstimulated cholesterol efflux (non-ABCA1 mediated) when all high HDL-C subjects, regardless of CAD status, were compared to subjects with normal HDL-C levels. It is possible that this difference is due to differences in the HDL particle number and size. Mutharasan et al. measured cholesterol efflux in a large cohort with a wide range of HDL values and found a positive relationship between cholesterol efflux capacity and HDL-C levels, HDL size, as well as HDL number and speculated that the cholesterol efflux capacity of HDL may be influenced by structural differences in HDL particles [40]. Indeed, Seckler et al. reported differences in apoA-I proteoforms in plasma from individuals with low and high cholesterol efflux capacity [41]. In addition, there may be differences in the lipid composition of HDL in subjects with high and normal HDL-C levels that could influence the cholesterol efflux capacity.

We found that the normolipidemic group had a significantly greater VLDL apoB100 PR than both groups of subjects with high HDL-C. Typically, the VLDL apoB100 PR is increased in obesity and in some type 2 diabetic subjects with hypertriglyceridemia [42]. However, the normolipidemic subjects were non-obese, and had normal fasting glucose levels and normal triglyceride levels. In a relatively large (*n* = 39) study previously conducted by our lab, the mean VLDL apoB100 PR was 13.30 ± 7.52 mg/kg/day (range 7.54–45.11 mg/kg/day) [43], which is similar to what we report for subjects with (14.5 mg/kg/day) and without (16.3 mg/kg/day) CAD in the current study. It is important to note that, while the normolipidemic subjects were significantly younger than the two groups of subjects with high HDL-C, VLDL apoB100 PR has been shown to increase with age so we do not believe age differences can explain this difference. Thus, the reason for the increase VLDL apoB100 production is not readily apparent and could be due to random selection of normal subjects with high VLDL apoB100 PR. On the other hand, we cannot rule out age being a factor responsible for other differences that we observed between the normolipidemic subjects and the two high HDL-C groups.

We performed an exploratory analysis to examine the relationships between in vivo HDL apoA-I and cholesterol kinetic parameters and ex vivo cholesterol efflux capacity. We found that the non-ABCA1 ex vivo cholesterol efflux capacity of HDL was significantly correlated with the HDL apoA-I PS and PR (i.e., subjects with higher apoA-I PS and PR have higher efflux capacity). We also found that the total and non-ABCA1 ex vivo cholesterol efflux capacities of HDL were significantly correlated with the HDL FC and CE PS (i.e., subjects with higher PS have higher efflux capacity) and inversely correlated with the HDL FC and CE pool FCR (i.e., HDL from subjects with rapid cholesterol turnover have lower efflux capacity). The correlations between the apoA-I PS and efflux may indicate that conditions in which there is a greater circulating number of HDL particles are associated with increased cholesterol efflux as measured by the ex vivo assay. Similarly, the correlation with HDL FC and CE PS may indicate greater total and non-ABCA1-mediated efflux in the presence of a greater number and larger particles. The inverse correlation between HDL cholesterol FCRs and cholesterol efflux is of interest and may relate to the HDL FC and CE PS. Since the HDL FC and CE FCR and corresponding PS are inversely correlated, it stands to reason that subjects with a high HDL FC and CE PS would have a high total and non-ABCA1 cholesterol efflux measurement but a low HDL FC and CE FCR. Conversely, subjects with a low HDL FC and CE PS would have a low total and non-ABCA1 cholesterol efflux measurement but a high HDL FC and CE FCR. This would imply that pool size may be the major determinant of ex vivo total and non-ABCA1-mediated efflux measurements. This possibility needs to be confirmed in a bigger number of subjects.

Our study has several limitations, including the small sample size and a limited normolipidemic reference group that was not matched by age. No significant differences were noted between subjects with elevated HDL-C levels with and without CAD, possibly due to the small sample size. However, our study supports the feasibility of dual labeling to simultaneously study the cholesterol and protein moieties of lipoproteins, and HDL specifically. Furthermore, this approach provided for the first time the opportunity to assess the association of ex vivo cholesterol efflux capacity with in vivo HDL cholesterol metabolic parameters. We observed that the apoA-I and HDL FC and CE PS and the HDL FC and CE FCRs are the best determinants of the total and non-ABCA1 cholesterol efflux capacity, suggesting that the efflux capacity may depend on the presence of HDL particles and the slow turnover of its cholesterol moieties.

## 4. Materials and Methods

### 4.1. Subjects

The protocol was first tested in a group of healthy normolipidemic controls with HDL within the normal range (*n* = 6) to optimize the study conditions. These subjects were recruited from lists of subjects that previously participated in clinical studies at the University of Pennsylvania or from IRB-approved advertisement. We then enrolled subjects with (*n* = 6) and without (*n* = 6) CAD that had HDL-C levels >90th percentile for age, gender, and race [44]. All subjects with elevated HDL-C had also participated in a larger lipidomic study [39]. We excluded subjects with plasma LDL-C levels greater than 160 mg/dL, triglycerides greater than 400 mg/dL, diabetes (type I and type II), history of liver disease with liver function tests greater than twice the upper limit of normal, history of kidney disease or chronic renal insufficiency, and use of medications known to significantly affect HDL-C levels (niacin, fibrates, fish oils, estrogen, testosterone or other steroids, cyclosporine or other immunosuppressants). Subjects with elevated HDL-C levels and CAD had documented cardiovascular disease defined as either a history of heart attack, angioplasty, coronary artery bypass surgery, coronary calcium score above the 90th percentile, or greater than 50% stenosis on CT angiogram. These subjects were matched with subjects with comparable elevated HDL-C levels, selected based on the absence of CAD and matched for race, gender, HDL-C level within 10 mg/dL, and to be the same age or up to 10 years older than the subjects with CAD. Additional exclusion criteria for these matched subjects included history of stroke, transient ischemic attack, and history of abdominal aortic aneurysm. The group of healthy normolipidemic controls was used as a reference.

### 4.2. Lipid and Apolipoprotein Measurements

Plasma concentrations of total cholesterol, HDL-C, triglycerides, and apolipoproteins were measured using blood samples obtained after a minimum of an 8-h fast using CDC-standardized methods. Plasma total cholesterol (TC), HDL-C, and triglycerides were measured enzymatically on a Cobas Fara II autoanalyzer (Roche Diagnostic Systems Inc., Basel, Switzerland) using Sigma reagents (Sigma Chemical Co. St Louis, MO, USA). Apolipoproteins were measured turbidimetrically using Wako reagents (Wako Chemicals USA Inc., Richmond, VA, USA). Measurements were performed on frozen (−80 °C) EDTA plasma and serum. LDL-C was calculated using the Friedewald equation.

### 4.3. Kinetic Study

Apolipoprotein and cholesterol kinetics were measured using an adaptation of two previously described protocols [45,46]. Briefly, participants were given a bolus of [5,5,5 D_3_]-leucine (10 µmol/kg) and [1,2-^13^C_2_]-acetate (4 or 240 µmol/kg) immediately followed by a constant infusion of [5,5,5-D_3_]-leucine (10 µmol/kg per hour) and [1,2-^13^C_2_]-acetate (4 or 240 µmol/kg per hour). Hourly standardized isocaloric meals were given throughout the 15 h kinetic study to maintain a constant rate of lipoprotein production. Meals contained 45% carbohydrate, 15% protein, and 40% fat. Blood samples were collected at various timepoints during and after the 15-h infusion period. Lipoprotein fractions were isolated from plasma by sequential ultracentrifugation. Briefly, VLDL, intermediate density lipoprotein (IDL), and LDL were isolated from 5 mL of plasma by ultracentrifugation at densities of 1.006, 1.019, and 1.063 g/mL, respectively, using a 50.2 Ti rotor (39,000 rpm for 18 h) (Beckman Instruments, Inc., Palo Alto, CA, USA). HDL was then isolated by adjusting the infranatant to a density of 1.21 g/mL and centrifuging at 39,000 rpm for a further 48 h [47].

For apolipoprotein kinetics, apolipoproteins were isolated by SDS-PAGE, hydrolyzed, their amino acids derivatized, and their isotope enrichment analyzed by GC/MS by the Metabolic Tracer Resource within the Institute of Diabetes, Obesity and Metabolism at Penn. The kinetics of VLDL apoB100 and HDL apoA-I and apoA-II were measured using WinSAAM by simultaneous fitting a multicompartmental model to the VLDL apoB100, HDL apoA-I, and apoA-II [5,5,5-D_3_]-leucine tracer data using a weighted least-squares approach to determine the best fit of the model to the tracer data as previously described [45].

For the HDL cholesterol kinetics, the HDL fraction was delipidated and free cholesterol and cholesteryl ester isolated from the lipid fraction by silica gel column chromatography. The cholesteryl ester fraction was then saponified and the resulting free cholesterol re-isolated by silica gel column chromatography. Cholesteryl ester fractions were saponified to release free cholesterol and enrichment of [^13^C] in free cholesterol from the free and cholesteryl ester fractions of HDL were determined using combustion/isotope ratio mass spectrometry (Metabolic Solutions, Nashua, NH). The kinetics of HDL free cholesterol and cholesteryl ester were measured by fitting a multicompartmental model to the corresponding enrichment data using a weighted least-squares approach to determine the best fit using WinSAAM. The precursor enrichment of [1,2-^13^C_2_]-acetate for cholesterol synthesis was assumed to be identical to that utilized for palmitate synthesis, which was determined using isotopomer spectral analysis of palmitate from VLDL [48]. Pool sizes (PSs) for free cholesterol and cholesteryl ester in HDL and nonHDL fractions were calculated as the plasma concentration (mg/dL) multiplied by plasma volume (0.45 dL/kg body weight). Production rates (PRs) were calculated using the formula PR (mg·kg^−1^·day^−1^) = [fractional clearance rate (FCR; pools per day) • concentration (mg/dL) • plasma volume (0.45 dL/kg body weight)]/body weight (kg).

### 4.4. Cholesterol Efflux

J774 mouse macrophage cells were plated and labeled with 2µCi of ^3^H-cholesterol/mL overnight. Cells were then incubated for 6 h in either the absence or presence of cyclic AMP (cAMP) to upregulate ABCA1. ApoB-containing lipoproteins were removed from serum by polyethylene glycol (PEG) precipitation. Efflux media containing the equivalent of 1% apoB-depleted serum (containing HDL) was incubated for 2 h at 37 °C. Media was collected and passed through a 0.22 μM filter prior to determining radioactivity by liquid scintillation counting after. A pooled plasma control was included on each plate to which samples from subjects were normalized. ABCA1-mediated cholesterol efflux capacity was determined by subtracting the basal cholesterol efflux capacity (without cAMP, non-ABCA1 cholesterol efflux) from the total cholesterol efflux capacity (with cAMP).

### 4.5. Statistical Analyses

Statistical analysis was performed using the programs Statistica 6 software (StatSoft, USA), GraphPad Prism 8, and R (Vienna, Austria, version 3.5.3). Discontinuous variables were analyzed using Fisher’s exact test. Data distributions were assessed for normality within subject groups using the Shapiro–Wilk test. The following variables were not-normally distributed in at least one group: VLDL apoB PS, VLDL apoB PR, HDL apoA-I PR, HDL FC and CE FCR, and HDL FC and CE PR. Normally distributed variables are displayed as the mean ± SD, while variables in which at least one group is not normally distributed are displayed as the median (interquartile range). Group comparisons for normally distributed variables were done using ANOVA followed by post-hoc Tukey HSD test. For variables with a non-normal distribution, comparisons were done using a Kruskal–Wallis test followed by post-hoc Dunn’s test. Correlations were examined using Spearman’s rank correlations. Values of *p* < 0.05 were considered statistically significant.

## Figures and Tables

**Table 1:** Age, sex, and plasma lipid and apolipoprotein data for subjects with high HDL with (+) and without (−) CAD as well as normolipidemic subjects. *n* = 6 for each group. Units for lipid and apolipoprotein parameters are mg/dL. Data are displayed as mean ± SD. Differences among groups were determined using ANOVA followed by a Tukey-HSD post-hoc test.

	High HDL CAD (+)	High HDL CAD (−)	Normolipidemic Reference
**Age (years)**	58 ± 8 **	65 ± 5 **	38 ± 14
**Sex (M/F)**	2/4	2/4	2/4
**BMI**	23.7 ± 2.0	25.7 ± 3.4	25.2 ± 2.7
**Total Cholesterol**	230 ± 46	236 ± 41	198 ± 47
**Triglyceride**	75 ± 18	70 ± 16	103 ± 51
**HDL-C**	108 ± 27 *	110 ± 29 *	64 ± 11
**LDL-C**	107 ± 39	107 ± 18	105 ± 31
**apoA-I**	230 ± 49	245 ± 46 *	171 ± 35
**apoA-II**	46 ± 8	43 ± 7	40 ± 11
**apoB**	82 ± 25	81 ± 13	76 ± 25

* *p* < 0.05; ** *p* < 0.01 vs. Normolipidemic Reference.

**Table 2:** VLDL apoB100 kinetic parameters in subjects with high HDL-C with and without CAD and normolipidemic subjects. *n* = 6 for each group. Normally distributed variables are displayed as mean ± SD, while variables in which at least one group is not normally distributed are displayed as the median (interquartile range). Differences among groups were determined using ANOVA followed by a Tukey-HSD post-hoc test for variables with a normal distribution and the Kruskal–Wallis test followed by a post-hoc Dunn’s test for variables with a non-normal distribution.

	High HDLCAD (+)	High HDL CAD (−)	Normolipidemic Reference
**VLDL ApoB100 PS (mg)**	194 (117–283)	401 (135–562)	288 (255–396)
**VLDL ApoB100 PR (mg/kg per day)**	17.3 (11.5–18.2) **	13.1 (7.85–24.2) *	28.3 (23.4–35.0)
**VLDL ApoB100 FCR (pools/day)**	(5.44 ± 2.24)	4.03 ± 2.20	6.32 ± 2.93

* *p* < 0.05; ** *p* < 0.01 vs. Normolipidemic Reference.

**Table 3:** Cholesterol efflux capacity of PEG-precipitated plasma for subjects with high HDL-C with and without CAD and normolipidemic subjects. Values are presented as percentage cholesterol effluxed from J774 cells over 6 h. N = 6 for High HDL CAD (−) and normolipidemic groups, *n* = 5 for High HDL CAD (+). Data are displayed as mean ± SD. Differences among groups were determined using ANOVA followed by a Tukey-HSD post-hoc test.

	High HDL CAD (+)	High HDL CAD (−)	Normolipidemic Reference
**% Total Efflux**	6.35 ± 2.19	6.39 ± 0.78	5.47 ± 0.96
**% Non-ABCA1-Mediated Efflux**	4.85 ± 1.93	5.29 ± 1.03	3.88 ± 0.87
**% ABCA1-mediated Efflux**	1.50 ± 0.43	1.10 ± 0.32	1.59 ± 0.82

**Table 4:** Spearman correlation coefficients between apoA-I and HDL cholesterol kinetic parameters and ex vivo cholesterol efflux for combined subject groups (*n* = 18). An asterisk (*) indicates *p* < 0.05, two (**) indicates *p* < 0.005, and three (***) indicates *p* < 0.0005.

	Total Efflux	Non-ABCA1 Mediated Efflux	ABCA1 Mediated Efflux
**apoA-I PS**	0.393	0.503 *	−0.052
**apoA-I PR**	0.348	0.552 *	−0.113
**apoA-I FCR**	−0.197	−0.057	0.037
**HDL FC PS**	0.836 ***	0.939 ***	−0.244
**HDL FC PR**	−0.056	0.157	−0.238
**HDL FC FCR**	−0.839 ***	−0.759 ***	0.146
**HDL CE PS**	0.726 **	0.796 ***	−0.358
**HDL CE PR**	−0.056	0.157	−0.238
**HDL CE FCR**	−0.779 ***	−0.652 **	0.112

## Data Availability

The data that support the findings of this study are available from the corresponding author upon reasonable request.

## Supplementary Materials

The following are available online. Figure S1: Scatterplots showing the relationships between selected HDL kinetic parameters and ex vivo cholesterol efflux.

## Sample Availability

Samples of compounds mentioned in this manuscript are not available from the authors.
