# Supplementary material for: Assessing HDL Metabolism in Subjects with Elevated Levels of HDL Cholesterol and Coronary Artery Disease
Source: Molecules. 2021 Nov 14;26(22):6862. doi: 10.3390/molecules26226862 (PMC8623898; doi:10.3390/molecules26226862)
Supplement: Supplementary file 1 [file molecules-26-06862-s001.zip › molecules-1379610-supplementary.pdf]

Supplementary Material

Assessing HDL Metabolism in Subjects with Elevated Levels of HDL Cholesterol and Coronary Artery Disease

William Hancock-Cerutti [1 †], John S. Millar [1], Silvia Valentini [1 ‡], Jason Liu [1], Jeffrey T. Billheimer [1], Daniel J. Rader [1] and Marina Cuchel [1,*]

1. Division of Translational Medicine and Human Genetics, Perelman School of Medicine University of Pennsylvania, Philadelphia, PA, USA; william.hancock-cerutti@yale.edu (W.H.-C.); jsmillar@pennmedicine.upenn.edu (J.S.M.); dottvalentini@gmail.com (S.V.); jasonyliumd@gmail.com (J.L.); billheij@pennmedicine.upenn.edu (J.T.B.); rader@pennmedicine.upenn.edu (D.J.R.)

† Yale School of Medicine, New Haven, CT, 06519 USA

‡ Unità Operativa Complessa Medicina e Chirurgia D'urgenza, Accettazione e Pronto Soccorso, Azienda Usl Toscana Sud Est, Grosseto, 58100 Italy

* Correspondence: mcuchel@pennmedicine.upenn.edu

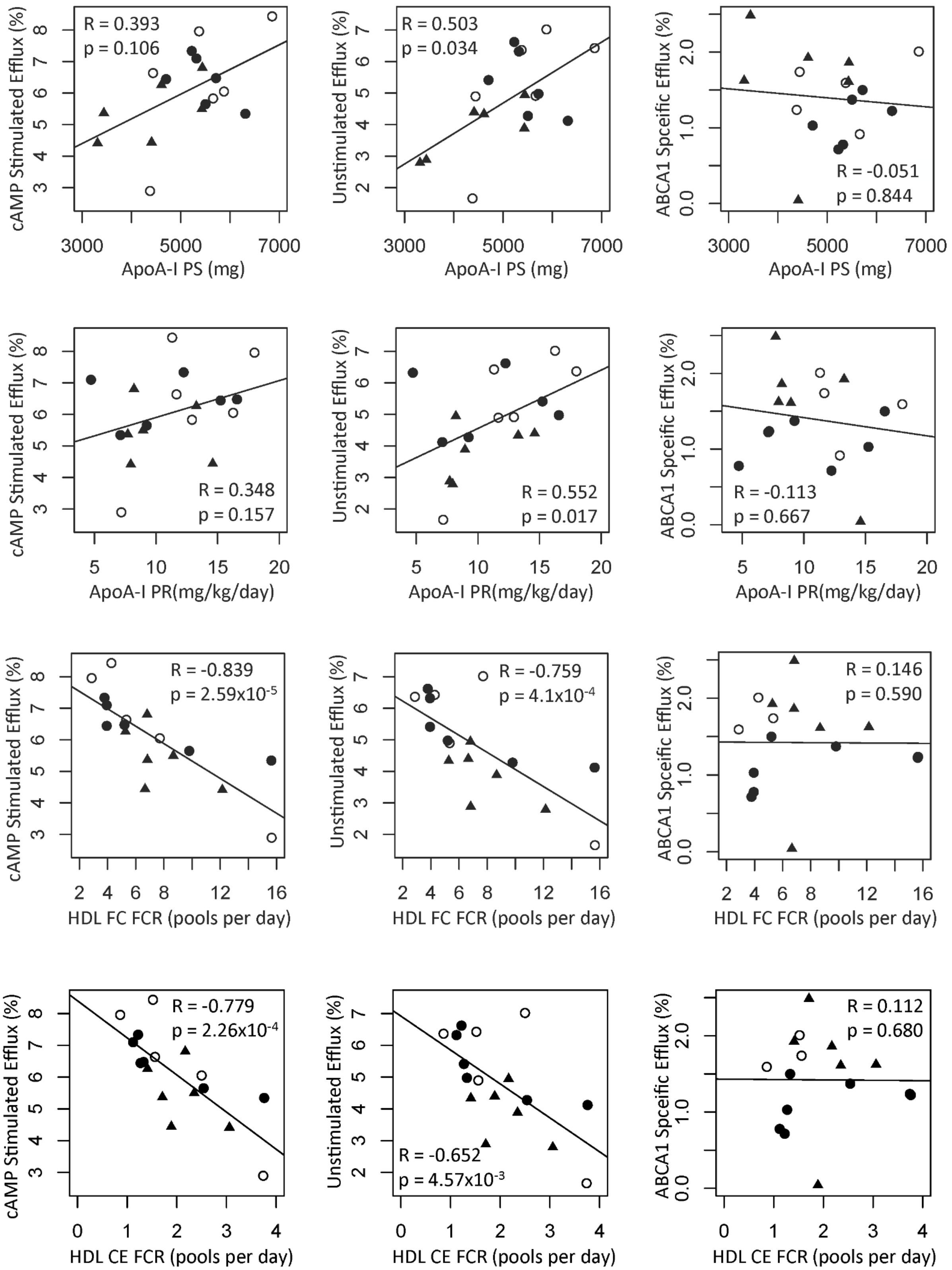

Figure S1. Scatterplots showing the relationships between selected HDL kinetic parameters and ex vivo cholesterol efflux. Empty circles denote High HDL CAD (+) subjects, filled circles denote High HDL CAD (−) subjects, and filled triangles denote normal controls. R = Spearman Correlation Coefficient.
